# Neutrophil-to-Lymphocyte and Platelet-to-Lymphocyte Ratios in Psoriatic Arthritis: A DAPSA-Based Validation Study

**DOI:** 10.31138/mjr.151205.ntl

**Published:** 2026-06-09

**Authors:** Cristina Macía-Villa, Mauro Ferre-Sanfrancisco, Ines Huerta-Tajuelo, Jose Luis Morell-Hita

**Affiliations:** Rheumatology Department, University Hospital Ramon y Cajal, Madrid, Spain

**Keywords:** psoriatic arthritis, disease activity, biomarkers, neutrophil-lymphocyte ratio, platelet-lymphocyte ratio, inflammation, C-reactive protein, receiver operating characteristic curve

## Abstract

**Objectives:**

Neutrophil-to-lymphocyte ratio (NLR) and platelet-to-lymphocyte ratio (PLR) are accessible markers of systemic inflammation. Their potential use as indicators of disease activity in psoriatic arthritis (PsA) remains unclear. This study aimed to validate NLR and PLR as markers of PsA activity using the Disease Activity in Psoriatic Arthritis (DAPSA) score as reference.

**Methods:**

This retrospective observational study included 426 PsA patients, with disease activity available for 102. Demographic, clinical, and laboratory data were retrieved from electronic records. Disease activity was assessed using DAPSA, with remission defined as ≤4 and active disease as ≥5. Discriminatory capacity was evaluated using receiver operating characteristic (ROC) curve analysis, including area under the curve (AUC), sensitivity, specificity, and predictive values.

**Results:**

Among the 102 patients with available DAPSA data, 39.2% were in remission and 60.8% had active disease. Mean NLR and PLR were 1.8 ± 0.6 and 104.5 ± 31.2 in remission versus 2.3 ± 2.8 and 134.7 ± 106.6 in active PsA (*p* = 0.311 and *p* = 0.081, respectively). ROC analysis showed poor discriminatory capacity for NLR (AUC 0.542, 95% CI 0.43–0.66, *p* = 0.47, sensitivity 68%, specificity 48%) and modest for PLR (AUC 0.604, 95% CI 0.50–0.71, *p* = 0.08, sensitivity 52%, specificity 57%), with optimal cut-offs of 1.55 and 111.1, respectively.

**Conclusions:**

NLR and PLR did not demonstrate sufficient validity as independent tools for classifying disease activity in PsA compared to DAPSA. Although their simplicity makes them attractive for clinical use, their diagnostic accuracy for PsA appears limited.

## INTRODUCTION

Neutrophil-to-lymphocyte ratio (NLR) and platelet-to-lymphocyte ratio (PLR) are simple, cost-effective, and easily accessible complete blood count (CBC)-derived markers that reflect systemic inflammation. Elevated values of these indices denote increased inflammatory activity and have been associated with a wide range of clinical conditions, including cancer, cardiovascular and metabolic diseases, infections, and auto-immune inflammatory disorders.^1^

Their biological plausibility in psoriatic disease (PsD) is supported by the interplay between the innate and adaptive immune responses. Psoriasis (PsO) and psoriatic arthritis (PsA) are characterised by dysregulated immune activation, with a shift in the balance between neutrophils, lymphocytes, platelets, and other cellular populations involved in systemic inflammation.^2^ Neutrophils infiltrate psoriatic lesions, release reactive oxygen species, and activate dendritic cells and Th17/Th22 lymphocyte subsets, promoting the secretion of cytokines such as IFN-γ, IL-17, IL-22, and TNF-α, which drive keratinocyte proliferation and chronic inflammation.^2–5^ Platelets actively participate in inflammation by releasing cytokines and interacting with immune cells.^6,7^ In contrast, lymphocytes are key regulators of adaptive immunity, and reduced lymphocyte counts may indicate immune dysregulation.^8^ Thus, NLR and PLR integrate these opposing cellular mechanisms, providing an indirect but dynamic measure of the systemic inflammatory burden.

Despite growing interest in these indices, a precise and unique cut-off value has not yet been defined. Reported normal ranges vary widely depending on sex, age, and ethnicity.^8–13^ In European healthy cohorts, the nor- mal NLR range has been reported between 0.78 and 3.53,^14,15^ while in Asian populations (predominantly Chinese cohorts) NLR values range from 0.88 to 4.0 and PLR values from 49 to 198.^9,11–13^ This variability underscores the need for population and disease specific validation before these markers can be reliably used in clinical practice.

In rheumatology, available evidence remains limited, with only two studies in rheumatoid arthritis (RA),^16,17^ one in gout,^18^ one on radiographic axial SpA (r-axSpA),1 and a single study specifically on PsA.^19^ However, most of these studies were exploratory or descriptive and did not formally evaluate the performance of NLR and PLR compared with validated composite indices of disease activity. Given this gap, further validation of NLR and PLR in PsA is required. Therefore, the objective of the present study was to validate NLR and PLR as indicators of disease activity in PsA, using the Disease Activity in Psoriatic Arthritis (DAPSA) score as the reference standard.

## MATERIAL AND METHODS

### Study design and patient population

We conducted a single-centre retrospective observational study including patients aged ≥18 years with PsA fulfilling the CASPAR classification criteria,^20^ evaluated at disease onset as part of routine clinical care from 2013 onwards. A total of 426 consecutive PsA patients were included for descriptive analyses. Disease activity assessment using DAPSA was available in a subcohort of 102 patients and was used for validation analyses.

### Patient, laboratory and disease activity variables

Demographic variables (age, sex, and body mass index), disease-related characteristics (age at PsO onset, age at PsA onset, delay between the two diseases, and clinical pattern of PsA), and treatment variables were collected from electronic medical records. Laboratory data obtained at the time of PsA diagnosis included C-reactive protein (CRP), NLR, and PLR, which were calculated as the ratios of neutrophils/lymphocytes and platelets/lymphocytes, respectively. Disease activity was evaluated using the DAPSA score. DAPSA data were available for a subset of patients (n = 102). For the purposes of this study, DAPSA was analysed as a binary variable, defining remission as ≤4 and active disease as ≥5. All laboratory determinations and disease activity assessment using DAPSA were performed at baseline, prior to the initiation of any conventional, targeted, or biologic DMARD therapy. Patients with acute infectious diseases, active malignancy, or other conditions known to significantly affect leukocyte or platelet counts at the time of blood sampling were excluded.

### Statistical analysis

Descriptive statistics were reported as mean ± standard deviation (SD) for continuous variables and as absolute values with percentages for categorical variables. Between-group comparisons were performed using the independent-samples Student’s *t*-test for continuous variables and the chi-square test for categorical variables. Correlations between continuous variables were assessed using Pearson’s correlation coefficient. Receiver operating characteristic (ROC) curve analysis was used to evaluate the discriminatory performance of NLR and PLR, using DAPSA remission versus active disease as the reference standard. The optimal cut-off values were determined according to the Youden index. Statistical significance was defined as a two-tailed p < 0.05. Normality was assessed using the Shapiro–Wilk test and visual inspection of distributions. If a variable showed mild skewness, results were confirmed using appropriate non-parametric tests (Mann–Whitney U test and Spearman’s correlation). Comparative analyses were performed between patients with and without available DAPSA data for demographic and clinical variables, and between remission and active disease groups for NLR and PLR among patients with available DAPSA data.

## ETHICAL APPROVAL

The study protocol was approved by the ethics committee of University Hospital Ramón y Cajal (reference 110/25).

## RESULTS

Demographic, clinical, and laboratory characteristics of the study population

A total of 426 patients with PsA were included in the study. The main demographic, clinical, and laboratory characteristics of the overall cohort and of the subgroup of patients with available DAPSA data (n = 102) are summarised in **Table 1**. In the overall cohort, 50.9% were women, with a mean body mass index of 28.1 ± 5.5 kg/m^2^. The mean age at PsO onset was 33.4 ± 17.2 years, and the mean age at PsA onset was 48.6 ± 12.7 years, with a median diagnostic delay of 12.5 years. Peripheral arthritis was the most frequent clinical pattern. Mean baseline values of NLR and PLR were 1.9 ± 1.3 and 116.9 ± 61.3, respectively.

**Table 1.** Baseline demographic, clinical, and laboratory characteristics of the overall psoriatic arthritis cohort and the available DAPSA subcohort.

	**Overall PsA cohort**	**DAPSA subcohort**	***p*** **value**

**Total patients**	426	102	NA

**Female sex, n** (%)	217 (50.9)	51 (50)	0.735

**BMI** (kg/m^2^)	28.1 ± 5.5	27.7 ± 4.8	0.310

**PsO age of onset** (years)	33.4 ± 17.2	29.8 ± 16.1	**0.031**
**PsA age of onset** (years)	48.6 ± 12.7	47.9 ± 13.1	0.350
**Delay between PsO and PsA onset** (years)	12.5 [0 – 62]	15.5 [0 – 59]	0.086

**PsA pattern**			
Peripheral, n (%)	385 (90.4)	99 (97.0)	**0.009**
Axial, n (%)	106 (24.9)	16 (15.7)	**0.014**
Mixed, n (%)	66 (15.5)	14 (13.7)	0.572

**NLR**	1.9 ± 1.3	2.1 ± 2.2	0.556
**PLR**	116.9 ± 61.3	122.6 ± 85.8	0.666
**CRP** (mg/L)	3.3 [0.2 – 84.6]	3.7 [0.2 – 32.7]	0.110
**DAPSA**	NA	4.8 [0 – 44.0]	NA
DAPSA remission (≤4)	NA	40 (39.2)	NA
DAPSA no remission (≥5)	NA	62 (60.8)	NA

**Treatment received**			
Number of csDMARDs	1.2 ± 0.7	1.4 ± 0.7	**0.001**
Number of ts/bDMARDs	0.9 ± 1.2	0.9 ± 1.2	0.561
Number of ts/bDMARDs targets	1.6 ± 0.8	1.6 ± 0.8	0.817

Quantitative variables are expressed as mean ± standard deviation (SD) or as median [range], as appropriate. Categorical variables are expressed as n (%). P-values correspond to comparisons between patients with available DAPSA data (n = 102) and those without available DAPSA data (n = 324), and p-values < 0.05 are shown in bold. BMI, body mass index. PsO, psoriasis. PsA, psoriatic arthritis. NLR, neutrophil-to-lymphocyte ratio. PLR, platelet-to-lymphocyte ratio. CRP, C-reactive protein. csDMARDs, conventional synthetic DMARDs. ts/bDMARDs, targeted/biologic DMARDs.

Disease activity assessment using DAPSA was available for 102 patients within the overall cohort and was used for subsequent analyses of disease activity and validation. Overall, patients with available DAPSA data showed broadly similar demographic and clinical characteristics compared with those without DAPSA assessment. Statistically significant differences between groups were observed for age at PsO onset, distribution of clinical patterns, and number of conventional synthetic DMARDs received (**Table 1**).

### Differences in NLR and PLR according to disease activity

When patients were stratified according to DAPSA status, both indices showed numerically lower values in the remission group than in the active disease group. Specifically, the mean NLR was 1.8 ± 0.6 in patients in remission and 2.3 ± 2.8 in those with active PsA (*p* = 0.311), while the mean PLR was 104.5 ± 31.2 versus 134.7 ± 106.6 (*p* = 0.081), respectively. Both indices were numerically higher in active disease, but the differences were not statistically significant. The data are summarised in **Table 2**.

**Table 2.** NLR and PLR levels based on disease activity.

	**DAPSA remission (≤4) n = 40**	**DAPSA no remission (≥5) n = 62**	***p*** **value**
**NLR**	1.8 ± 0.6	2.3 ± 2.8	0.311
**PLR**	104.5 ± 31.2	134.7 ± 106.6	0.081

NLR neutrophil/lymphocyte ratio. PLR platelet/lymphocyte ratio.

### ROC analysis and diagnostic performance of NLR and PLR

**Figure 1.**
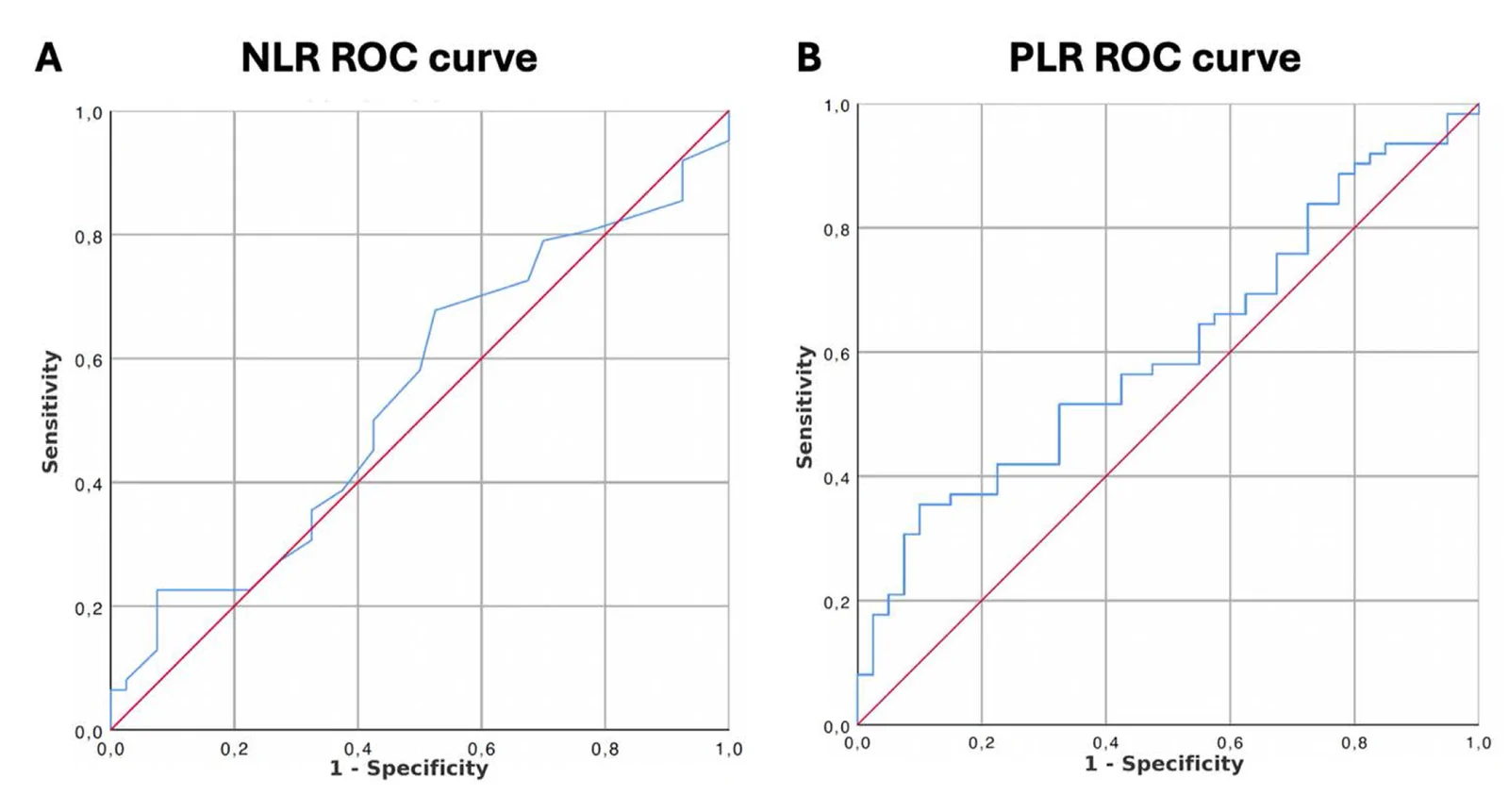
Receiver operating characteristic (ROC) curves of NLR and PLR for discriminating disease activity in psoriatic arthritis according to DAPSA. Receiver operating characteristic (ROC) curves illustrating the performance of the neutrophil-to-lymphocyte ratio (NLR) and the platelet-tolymphocyte ratio (PLR) in classifying patients according to disease activity status, using DAPSA remission (≤4) versus active disease (≥5) as the reference standard. Panel A shows the ROC curve for NLR and panel B for PLR. Sensitivity is shown on the y-axis and 1 – specificity on the x-axis.

ROC curve analysis showed that NLR had a poor discriminatory capacity for disease activity (**Figure 1**), with an AUC of 0.542 (95% CI 0.428–0.657, *p* 0.472). The best cut-off (1.55) yielded 68% sensitivity and 48% specificity, with a PPV of 67%, NPV of 49%, LR+ of 1.31, and LR– of 0.67 (**Table 3**). PLR showed modest performance (**Figure 1**), with an AUC of 0.604 (95% CI 0.495– 0.714, *p* 0.076). At the cut-off of 111.1, the sensitivity was 52% and specificity was 57%, with PPV of 65%, NPV of 43%, LR+ of 1.21, and LR– of 0.84 (**Table 3**).

**Table 3.** Diagnostic performance of neutrophil-to-lymphocyte ratio (NLR) and platelet-to-lymphocyte ratio (PLR) for identifying PsA remission (DAPSA ≤4) versus active disease (DAPSA ≥5).

**Variable**	**AUC 95% CI**	**Cut-off value**	**Sensitivity**	**Specificity**	**PPV**	**NPV**	**LR+**	**LR−**	***p*** **value**
**NLR**	0.542 (0.428–0.657)	1.55	0.68	0.48	0.67	0.49	1.31	0.67	0.472
**PLR**	0.604 (0.495–0.714)	111.1	0.52	0.57	0.65	0.43	1.21	0.84	0.076

NLR neutrophil/lymphocyte ratio. PLR platelet/lymphocyte ratio. AUC area under the ROC curve. CI confidence interval. PPV positive predictive value. NPV negative predictive value. LR+ positive likelihood ratio. LR– negative likelihood ratio.

## DISCUSSION

The NLR and PLR are simple and easily accessible indices that can be obtained from routine CBC. This study aimed to validate these two haematological indices as potential markers of disease activity in PsA, using the DAPSA score as the reference standard. Both indices in our cohort demonstrated only modest discriminatory capacity, with areas under the ROC curve close to 0.5, and neither significantly outperformed the DAPSA in classifying patients into remission or active disease. The identified cut-off values (1.55 for NLR and 111.1 for PLR) were lower than those reported for other rheumatic diseases such as RA^16^ and gout,^18^ but similar to those described for r-axSpA.^1^ This difference may reflect the underlying pathophysiological difference between rheumatic diseases. In SpA and PsD, inflammation is driven by the IL-23/Th17 axis and shows less systemic neutrophilic activation than in RA or gout, in which acute-phase responses are more intense. This could explain the lower NLR and PLR thresholds observed in PsA and supports the need for disease-specific validation of these indices.^2,3, 5,8^

In immune-mediated diseases, NLR and PLR are considered markers of systemic inflammation and tend to show higher values compared with healthy populations.^1,3–5,16–18,21,22^ They also decreased following specific and effective treatments.^6,7,22–25^ However, available evidence on PsD remains limited. Most studies have focused on PsO,^2,4,7, 21,22, 25^ with only small subsets of patients with concomitant PsA included in their cohorts.^3, 5,6,23,24^ To date, only one study has specifically examined NLR and PLR in PsA,^19^ but our study is the first to validate their diagnostic performance against DAPSA using ROC curve analysis.

Across studies on axSpA and PsD, NLR and PLR have consistently shown only a weak association with traditional inflammatory markers such as CRP,^4,6,7,23,24^ and overall, these indices do not outperform established composite measures of disease activity. Similar findings have been reported in r-axSpA, where both ratios showed weak correlations with disease activity scores and limited diagnostic validity.^1^ In PsA, our results are consistent with those described by Keleşoğlu Dinçer et al.,^19^ who also found only modest correlations between NLR, PLR, and DAPSA scores. In our cohort, neither index reached statistical significance, and both demonstrated poor discriminatory ability in the ROC analysis. Although PLR values tended to be higher in patients with active PsA, this difference narrowly missed statistical significance (*p* = 0.08), suggesting a possible subclinical trend that might become evident in larger or longitudinal studies in the future. In PsO, the evidence remains inconsistent. Three recent meta-analyses^2,3,21^ yielded conflicting results, with only one^3^ reporting a significant association between disease severity and these indices. Collectively, available data indicate that NLR and PLR, like CRP, reflect nonspecific systemic inflammation rather than disease-specific activity, being influenced by age, sex, ethnicity, and comorbidities.^8^

Although the DAPSA is currently the most widely used composite index for assessing disease activity in PsA, it cannot be considered an absolute gold standard. Indeed, there is no universally accepted benchmark against which to validate alternative inflammatory markers such as NLR or PLR, and DAPSA or other validated scores simply represent the best reference tools available. Moreover, in daily clinical practice, the calculation of these composite indices may be limited by time constraints or incomplete data, which justifies the interest in exploring simpler and more readily available laboratory parameters derived from routine blood counts. In this context, NLR and PLR offer practical, low-cost alternatives for assessing systemic inflammation and may provide complementary information within a broader assessment of the patient.

Although patients with available DAPSA data showed some statistically significant differences compared with those without DAPSA assessment, including an earlier age at PsO onset, a higher prevalence of peripheral disease, a lower frequency of axial involvement, and a greater number of csDMARDs received, these differences likely reflect patterns of clinical assessment in routine practice rather than true disease heterogeneity. Importantly, baseline NLR and PLR values, the primary variables of interest in this study, were comparable between groups, and the absolute differences observed in other variables were modest. Therefore, these findings are unlikely to have influenced the central analysis or to represent relevant confounding factors in the evaluation of NLR and PLR as markers of disease activity.

This study has some limitations inherent to its design. It was a retrospective, single-centre analysis, and the number of patients with complete DAPSA data was limited, which may restrict the generalisability of the findings. In addition, although NLR and PLR are known to vary according to demographic and disease-related factors, subgroup analyses stratified by sex and ethnicity were not performed due to the balanced sex distribution and the predominantly Caucasian population of our cohort, while age-stratified analyses were beyond the scope of the present study, which focused on the evaluation of NLR and PLR in relation to disease activity in PsA. Despite these constraints, the study has notable strengths, including the use of a validated composite index as a reference standard, a robust analytical approach based on ROC curves analysis, and, to our knowledge, the first focused attempt to validate NLR and PLR as markers of disease activity specifically in PsA.

In conclusion, NLR and PLR did not demonstrate sufficient validity as independent tools for classifying disease activity in PsA compared with DAPSA. Nevertheless, their simplicity, low cost, and accessibility justify the continued interest in using these indices as potential complementary markers. It is conceivable that their performance could differ in prospective, multicentre studies specifically designed to account for the known variability of these ratios across age, sex, ethnicity, and between different immune-mediated diseases, using validated disease-specific composite indices as reference standards. Such studies would help refine the interpretation of the NLR and PLR in rheumatic diseases and determine whether they may have a more defined role in future clinical practice.
